# EphA4-ADAM10 Interplay Patterns the Cochlear Sensory Epithelium through Local Disruption of Adherens Junctions

**DOI:** 10.1016/j.isci.2018.12.017

**Published:** 2018-12-26

**Authors:** Jean Defourny, Christiane Peuckert, Klas Kullander, Brigitte Malgrange

**Affiliations:** 1 GIGA-Neurosciences, Unit of Cell and Tissue Biology, University of Liège, C.H.U. B36, 4000 Liège, Belgium; 2 GIGA-Neurosciences, Developmental Neurobiology Unit, University of Liège, C.H.U. B36, 4000 Liège, Belgium; 3 Department of Neuroscience, Uppsala University, Box 593, Uppsala 75124, Sweden

**Keywords:** Physiology, Cell Biology, Developmental Biology

## Abstract

The cochlear sensory epithelium contains a functionally important triangular fluid-filled space between adjacent pillar cells referred to as the tunnel of Corti. However, the molecular mechanisms leading to local cell-cell separation during development remain elusive. Here we show that EphA4 associates with ADAM10 to promote the destruction of E-cadherin-based adhesions between adjacent pillar cells. These cells fail to separate from each other, and E-cadherin abnormally persists at the pillar cell junction in EphA4 forward-signaling-deficient mice, as well as in the presence of ADAM10 inhibitor. Using immunolabeling and an *in situ* proximity ligation assay, we found that EphA4 forms a complex with E-cadherin and its sheddase ADAM10, which could be activated by ephrin-B2 across the pillar cell junction to trigger the cleavage of E-cadherin. Altogether, our findings provide a new molecular insight into the regulation of adherens junctions, which might be extended to a variety of physiological or pathological processes.

## Highlights

• EphA4 and ephrin-B2 are co-expressed on both sides of the pillar cell junction
• Pillar cells fail to separate from each other in the absence of EphA4 signaling
• EphA4 forms a complex with E-cadherin and ADAM10 at the pillar cell junction
• E-cadherin inhibition rescues the pillar cell detachment in the absence of EphA4

## Graphical Abstract

**Figure:**
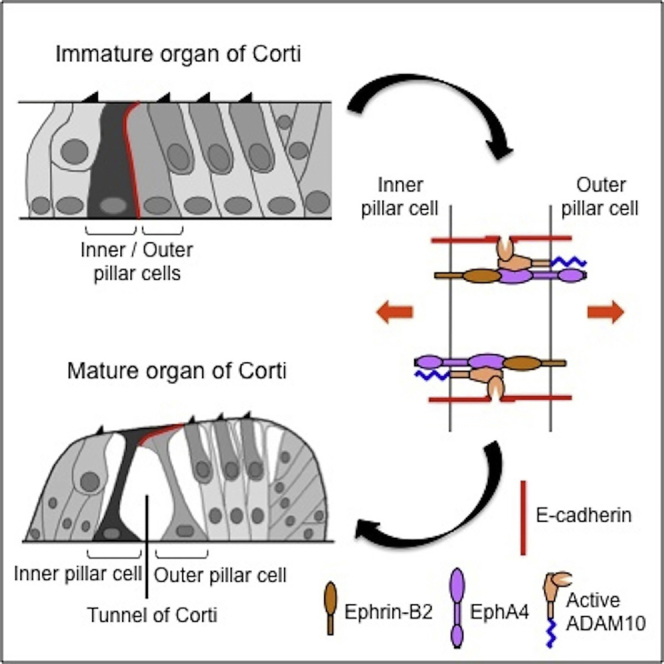

## Introduction

**Figure 1:**
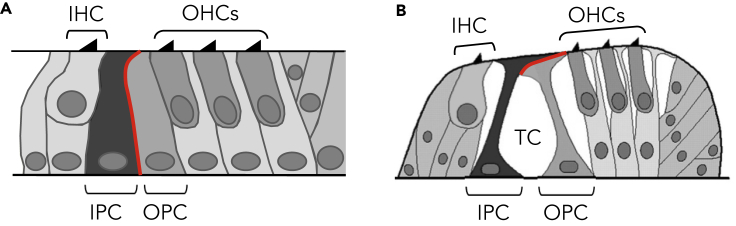
Schematic Representation of E-Cadherin Localization between Immature and Mature Adjacent IPC and OPC (A) At the immature stage, all the cells of the organ of Corti are closely connected and the IPCs abut on the OPCs through E-cadherin-based homophilic adhesions (in red). (B) When mature, the IPCs and OPCs form the boundaries of a triangular fluid-filled space and only the apical extremities of the PCs are still connected through E-cadherin adhesions (in red). IHC, inner hair cell; IPC, inner pillar cell; OHC, outer hair cell; OPC, outer pillar cell; TC, tunnel of Corti.

In mammals, sounds are perceived through mechanosensory hair cells located in the sensory epithelium of the cochlea (the organ of Corti). Within the organ of Corti, hair cells and several types of specialized supporting cells are arranged in a regular mosaic pattern that extends along the basal-to-apical axis of the cochlear duct. The organ of Corti contains one row of inner hair cells and three rows of outer hair cells separated by two parallel rows of non-sensory pillar cells (PCs). At birth, all cells of the organ of Corti are closely connected and the inner PCs (IPCs) abut on the adjacent outer PCs (OPCs) through E-cadherin-based homophilic adhesion interactions (Johnen et al., 2012, Whitlon, 1993) (red curve in Figure 1A). When mature, i.e., from 2 weeks of age (postnatal day 14, P14) in mice, these rows of PCs form the boundaries of a functionally important triangular fluid-filled space referred to as the tunnel of Corti (Raphael and Altschuler, 2003) (Figure 1B). Previous studies have suggested that proper development of the tunnel of Corti is required for hearing (Chen and Segil, 1999, Colvin et al., 1996, Inoshita et al., 2008). Indeed, the somatic motility of outer hair cells produces oscillatory fluid flow in the tunnel of Corti, which is critical for cochlear amplification (Karavitaki and Mountain, 2007). The molecules underlying PC fate acquisition are now well characterized and include fibroblast growth factors and their receptors (Colvin et al., 1996, Doetzlhofer et al., 2009, Jacques et al., 2007, Mueller et al., 2002). In contrast, the mechanisms leading to local IPC/OPC separation remain elusive. However, a critical step should be the progressive loss of the adhesion protein E-cadherin from the lateral membranes of the PCs (Johnen et al., 2012, Whitlon, 1993) (Figure 1).

An attractive candidate for this process is Eph/ephrin signaling, which has been shown to control several aspects of cochlear development (Defourny et al., 2013, Defourny et al., 2015). The large Eph receptor family has been classified into two subclasses, EphA and EphB receptors, according to their affinity for either glycosylphosphatidylinositol-anchored ephrin-A or transmembrane ephrin-B ligands (Gale et al., 1996). However, some cross-class interactions are possible, as EphA4 can also bind to ephrin-B ligands (Bowden et al., 2009, Qin et al., 2010). Upon binding, receptor clustering initiates a “forward signaling” but receptor-ligand interaction can also stimulate a “reverse signaling” downstream of the ephrin ligand (Kullander and Klein, 2002). The special case of antiparallel signaling occurs when ephrin and Eph are co-expressed on two apposed cells and result in Eph receptor forward signaling in both directions (Kania and Klein, 2016, Rohani et al., 2011). During organogenesis, ephrin and Eph receptor genes control a wide range of critical processes such as cell sorting and positioning, and the formation of segmented structures (Kania and Klein, 2016). Among a broad variety of pathways, EphB/ephrin-B signaling has been shown to dictate the destruction of E-cadherin-based adhesions in the intestinal epithelium (Solanas et al., 2011). Beside EphB receptors, EphA4 also affects the expression of E-cadherin in cancer cells (de Marcondes et al., 2016, Liu et al., 2014), suggesting this protein as a good candidate for patterning the tunnel of Corti through local downregulation of adherens junctions.

Here we show that EphA4 and its ligand ephrin-B2 are each expressed on both sides of the IPC/OPC junction. These adjacent PCs fail to separate from each other, and E-cadherin abnormally persists at the PC junction in EphA4 forward-signaling-deficient mice, as well as in the presence of ADAM10 inhibitor. By combining immunolabeling and an *in situ* proximity ligation assay, we found that EphA4 forms a complex with E-cadherin and its sheddase ADAM10, which could be activated by ephrin-B2 across the PC junction to trigger the cleavage of E-cadherin. Altogether, our results highlight a key role for EphA4-ADAM10 interplay in patterning the cochlear sensory epithelium.

## Results

### EphA4 and Ephrin-B2 Are Co-expressed on Both Sides of the IPC/OPC Junction

**Figure 2:**
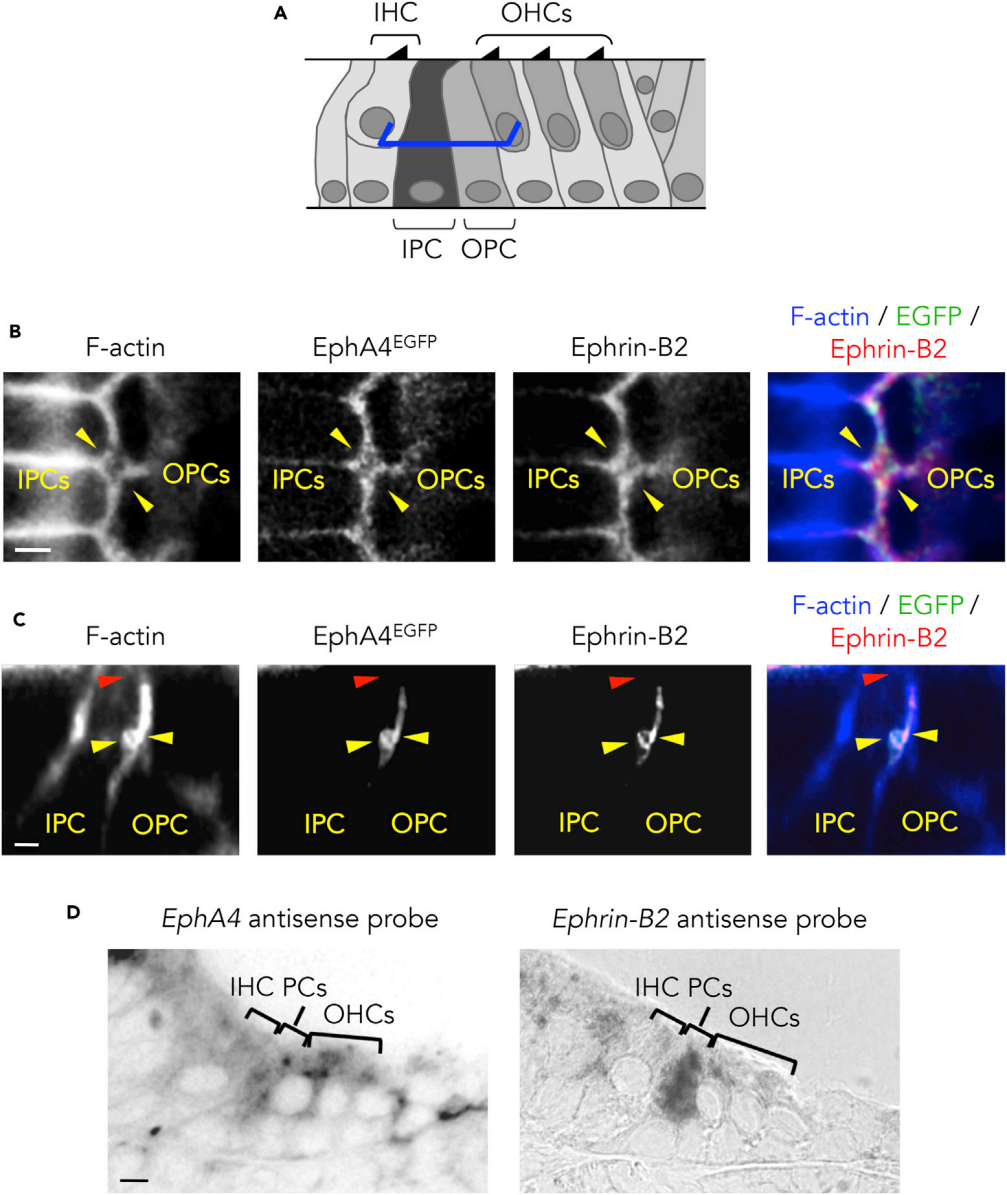
EphA4 and Ephrin-B2 Are Co-expressed on Both Sides of the IPC/OPC Junction (A) Schematic representation of a P4 mouse organ of Corti showing the position at which level single confocal images were acquired (here at half-height of PCs, blue segments). (B) F-actin staining and ephrin-B2 immunolabeling of P4 *EphA4*^*+/EGFP*^ whole-mount cochlea showing that EphA4^EGFP^ and ephrin-B2 are co-expressed on both sides of the IPC/OPC junction (yellow arrowheads). (C) Orthogonal projection of P4 *EphA4*^*+/EGFP*^ whole-mount PCs showing that EphA4^EGFP^ and ephrin-B2 are reciprocally co-expressed at about half-height of the PC junction (yellow arrowheads). In contrast, neither EphA4^EGFP^ nor ephrin-B2 is expressed at the apical extremity of the PCs, i.e., where the PCs remain closely connected (red arrowhead). (D) *In situ* hybridization on transversal section of P4 cochlea showing that *EphA4* and *ephrin-B2* are both expressed in the PCs. See also Figures S1 and S2. Scale bars, 2 μm in (B) and (C) and 5 μm in (D). IHC, inner hair cell; IPC, inner pillar cell; OHC, outer hair cell; OPC, outer pillar cell; PC, pillar cell.

At immature stages, all cells of the organ of Corti are closely connected and the IPCs abut on the OPCs (Figure 1A). As development progresses, the apical ends of the PCs remain connected, forming the reticular lamina, whereas the lateral membranes become no longer apposed, being separated with fluid spaces (Figure 1B). This process suggests a subcellular mechanism occurring at about half-height of the PC junction to promote a local IPC/OPC detachment. Among the Eph and ephrin families, EphA4 and its ligand ephrin-B2 are frequently involved in cell repulsion and tissue segmentation processes (Mellitzer et al., 1999, Xu et al., 1999), including in the developing cochlea (Defourny et al., 2015). Therefore, we examined their expression patterns during the postnatal development of the tunnel of Corti. By using an *EphA4*^*+/EGFP*^ reporter mouse model (Grunwald et al., 2004) combined with ephrin-B2 immunolabeling, we found that EphA4^EGFP^ and ephrin-B2 are co-expressed on both sides of the IPC/OPC junction from postnatal day 4 (P4) (Figures 2B and 2C), i.e., when the early opening of the tunnel of Corti is initiated (Ito et al., 1995). Importantly, these expression patterns are spatially restricted to the half-height region of the PCs. At postnatal stages, neither EphA4^EGFP^ nor ephrin-B2 is expressed at the apical extremities of the PCs, which remain connected in the mature configuration of the tunnel of Corti (Figures 2C and S1). The presence of EphA4 and ephrin-B2 in the PCs from P4 was further confirmed using *in situ* hybridization (Figure 2D). The corresponding negative controls were obtained using sense probes (Figure S2). These overlapping expression patterns across the IPC/OPC boundary are consistent with findings suggesting that two Eph/ephrin antiparallel forward signals are sufficient to regulate cell-cell detachment. In this case, each pathway involves ephrin ligands on one side and Eph receptors on the other side (Rohani et al., 2011).

### IPCs and OPCs Fail to Separate from Each Other in EphA4^EGFP/EGFP^ Mice

**Figure 3:**
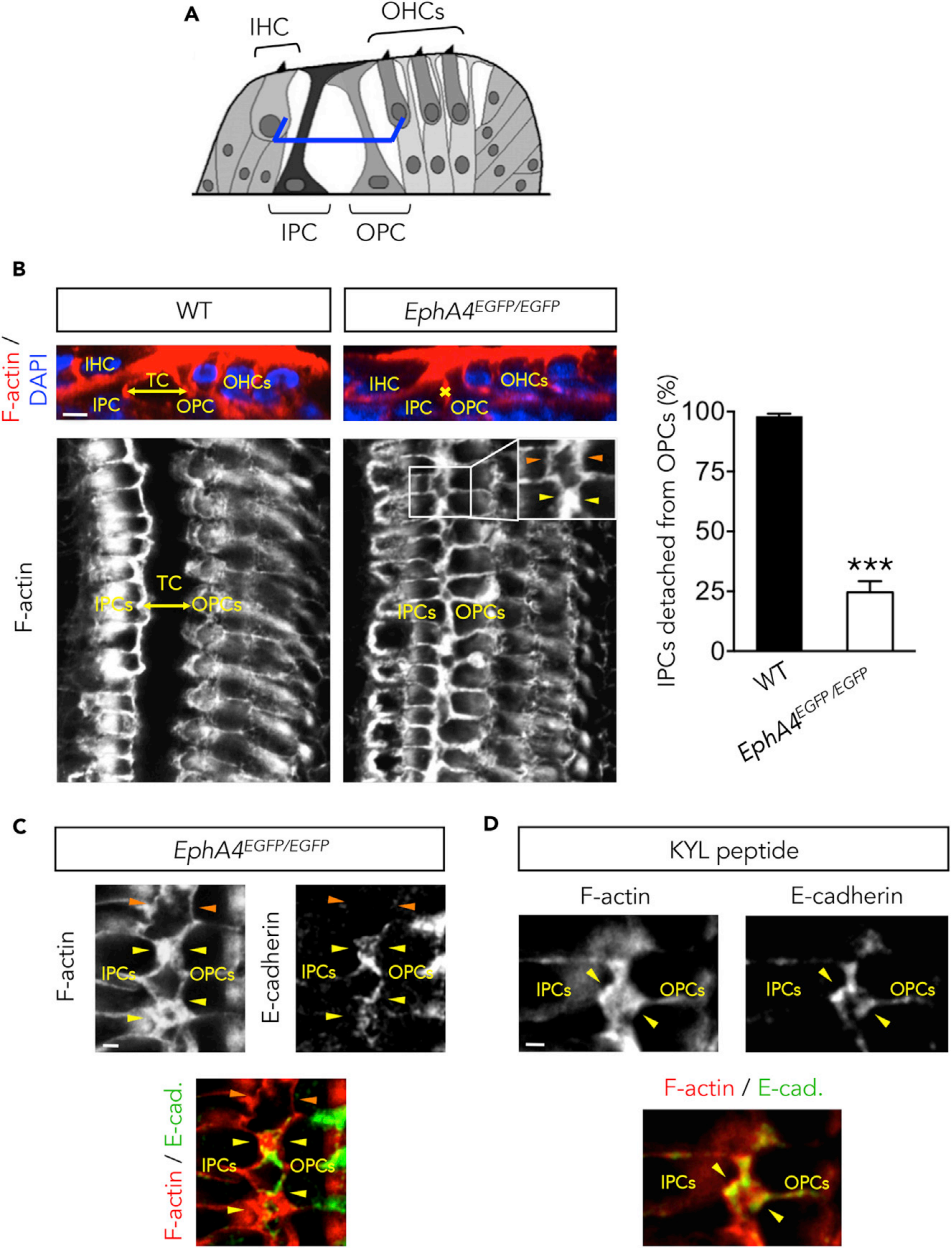
IPCs and OPCs Fail to Separate from Each Other in the Absence of EphA4 Signaling (A) Schematic representation of a mature (P14) organ of Corti showing the position at which level single confocal images were acquired (here at half-height of PCs, blue segments). (B) F-actin and DAPI nuclear staining of P14 WT and *EphA4*^*EGFP/EGFP*^ whole-mount cochleae. Upper panels: orthogonal reconstruction of confocal images showing the presence (double-headed arrow) or the absence (asterisk) of a triangular space between the IPC and OPC in WT or *EphA4*^*EGFP/EGFP*^ cochlea, respectively. Lower panels: confocal images showing that, in WT mice, all the IPCs are detached from the OPCs, allowing the formation of the tunnel of Corti (double-headed arrow). In *EphA4*^*EGFP*^^*/EGFP*^ mice, a few IPCs are fully detached from the adjacent OPCs (inset, orange arrowheads) and a majority of PCs remain attached to each other (inset, yellow arrowheads). Right panel: the percentage of IPCs detached from OPCs is significantly decreased in *EphA4*^*EGFP/EGFP*^ when compared with WT mice. n = 200 IPCs from four animals of both genotypes. (C) F-actin staining and E-cadherin immunolabeling of P14 *EphA4*^*EGFP/EGFP*^ whole-mount cochlea showing that E-cadherin abnormally persists at the IPC/OPC junction (yellow arrowheads), whereas it is absent from separated membranes (orange arrowheads). (D) F-actin staining and E-cadherin immunolabeling of organotypic culture treated with KYL peptide showing that E-cadherin persists at the IPC/OPC junction (yellow arrowheads). Data are presented as mean ± SEM. ***p < 0.001. See also Figure S3. Scale bars, 5 μm in (B) and 2 μm in (C) and (D). IHC, inner hair cell; IPC, inner pillar cell; OHC, outer hair cell; OPC, outer pillar cell; TC, tunnel of Corti.

Eph forward signaling rather than ephrin reverse signaling has been shown to dictate the destruction of E-cadherin-based adhesions (Solanas et al., 2011) and to promote cell-cell separation (Rohani et al., 2011). To test whether EphA4 forward signaling promotes IPC/OPC detachment, we compared the proportion of IPCs that are fully detached from the adjacent OPCs in whole-mount cochleae from P14 wild-type (WT) and knockin mice encoding an EphA4 forward-signaling-deficient isoform (Grunwald et al., 2004). We found that the percentage of IPCs entirely detached from the adjacent OPCs at half-height of the organ of Corti is significantly decreased in the absence of the EphA4 cytoplasmic domain (Figure 3B). As a consequence, the classic triangular shape of the tunnel of Corti observed in WT mice failed to correctly develop in *EphA4*^*EGFP/EGFP*^ animals (Figure 3B). Such defects were found in 75% of the knockin mice (n = 9 out of 12) and in none of the WT mice (n = 8). This level of penetrance in *EphA4*^*EGFP/EGFP*^ mice is similar to previously published data (Egea et al., 2005, Peuckert et al., 2016). In addition, we found that E-cadherin abnormally persists at the IPC/OPC junctions in P14 *EphA4*^*EGFP/EGFP*^ mice (Figure 3C), suggesting that EphA4 forward signaling downregulates E-cadherin to promote PC detachment. Of note, no earlier defects regarding the PCs were observed in P2 *EphA4*^*EGFP/EGFP*^ mice (Figure S3). The role of EphA4 in the local disruption of adherens junctions was further confirmed in organotypic culture using KYL peptide, a specific inhibitor of EphA4 (Lamberto et al., 2012) (Figure 3D).

### EphA4 Forms a Complex with E-Cadherin and ADAM10 at the IPC/OPC Junction

**Figure 4:**
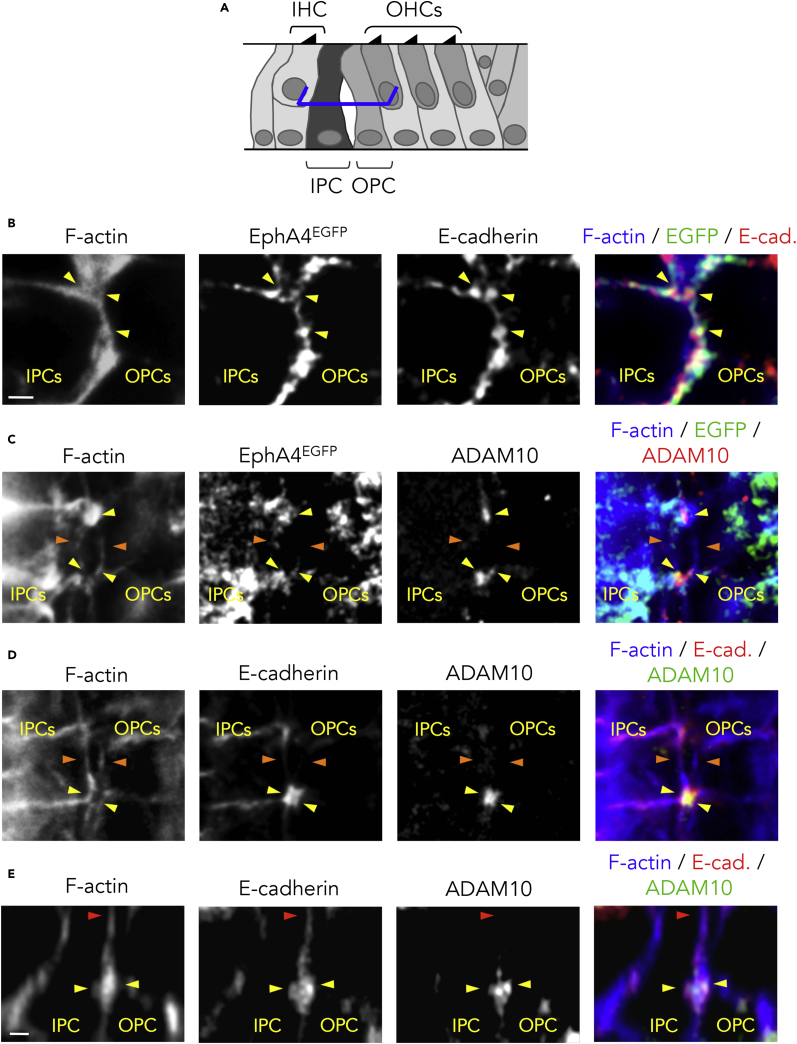
EphA4 Co-localizes with E-Cadherin and ADAM10 at the IPC/OPC Junction (A) Schematic representation of a P6 mouse organ of Corti showing the position at which level single confocal images were acquired (here at half-height of PCs, blue segments). (B) F-actin staining and E-cadherin immunolabeling of P6 *EphA4*^*+/EGFP*^ whole-mount cochlea showing that EphA4^EGFP^ and E-cadherin co-localize on both sides of the IPC/OPC junction (yellow arrowheads). (C) F-actin staining and ADAM10 immunolabeling of P6 *EphA4*^*+/EGFP*^ whole-mount cochlea showing that EphA4^EGFP^ and ADAM10 co-localize on both sides of the IPC/OPC junction (yellow arrowheads) and disappear as soon as the membranes are detached from each other (orange arrowheads). (D) F-actin staining, E-cadherin, and ADAM10 immunolabeling of P6 WT whole-mount cochlea showing that E-cadherin and ADAM10 co-localize on both sides of the IPC/OPC junction (yellow arrowheads) and disappear as soon as the membranes are detached from each other (orange arrowheads). (E) Orthogonal projection of P6 WT whole-mount PCs showing that E-cadherin and ADAM10 are reciprocally co-expressed at about half-height of the PC junction (yellow arrowheads). In contrast, E-cadherin but not ADAM10 is present at the apical extremity of the PC junction, i.e., where the PCs remain closely connected (red arrowhead). See also Figure S4. Scale bars, 2 μm in (B–D) and (E). IHC, inner hair cell; IPC, inner pillar cell; OHC, outer hair cell; OPC, outer pillar cell.

**Figure 5:**
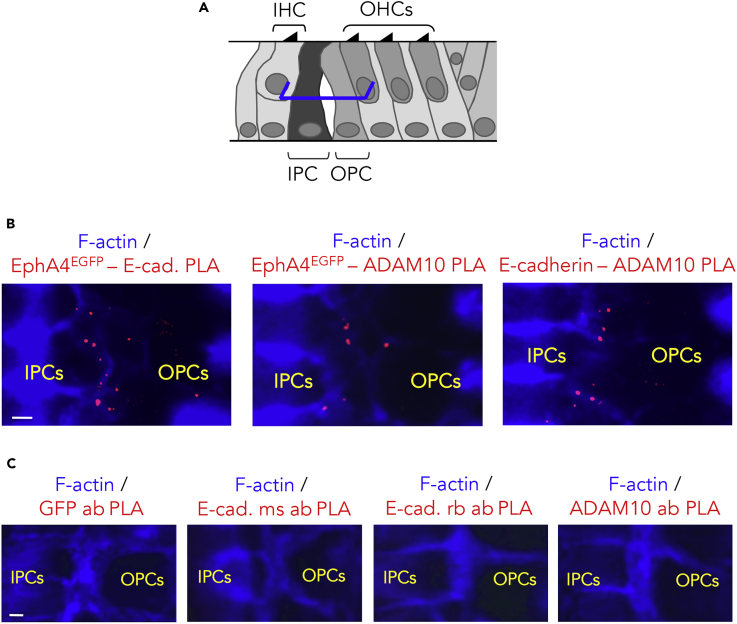
EphA4 Forms a Complex with E-Cadherin and ADAM10 at the IPC/OPC Junction (A) Schematic representation of a P6 mouse organ of Corti showing the position at which level single confocal images were acquired (here at half-height of PCs, blue segments). (B) *In situ* proximity ligation assay showing that EphA4^EGFP^ interacts with E-cadherin and ADAM10, as well as E-cadherin with ADAM10 at half-height of the IPC/OPC junction. Protein-protein interactions are detected as fluorescent red spots. (C) Proximity ligation assay negative controls were performed using a single primary antibody (ab): anti-GFP, mouse, or rabbit anti-E-cadherin and anti-ADAM10. See also Figures S5 and S6. Scale bars, 2 μm in (B) and (C). IHC, inner hair cell; IPC, inner pillar cell; OHC, outer hair cell; OPC, outer pillar cell; PLA, proximity ligation assay.

To decipher the molecular mechanisms through which EphA4 downregulates the adherens junctions in the cochlea, we examined whether EphA4^EGFP^ associates with E-cadherin and ADAM10. This disintegrin and metalloproteinase promotes the shedding of E-cadherin (Maretzky et al., 2005) and constitutively associates with Eph receptors via specific regions in their extracellular domains (Janes et al., 2005). From P6, some fluid-filled spaces begin to intersperse the two rows of adjacent PCs (Ito et al., 1995). At this stage, we found that, at half-height of the PCs, E-cadherin (yellow arrowheads in Figure 4B) and ADAM10 (yellow arrowheads in Figure 4C) co-localized with EphA4^EGFP^ at the IPC/OPC junction in *EphA4*^*+/EGFP*^ mice, and with ADAM10 in WT mice (yellow arrowheads in Figures 4D and 4E). Concomitantly, we observed that EphA4^EGFP^ (orange arrowheads in Figure 4C), E-cadherin (orange arrowheads in Figure 4D), and ADAM10 (orange arrowheads in Figures 4C and 4D) disappeared as soon as the membranes were detached from each other, suggesting that these three proteins are closely involved in cell-cell separation. In contrast, the expression of E-cadherin was maintained at apical extremities of the PCs, as expected (Johnen et al., 2012, Whitlon, 1993) (red arrowhead in Figures 4E and S4). Similarly to EphA4^EGFP^, ADAM10 was not found at the apical ends of PCs, i.e., where the IPCs and OPCs remain attached to each other (red arrowhead in Figures 4E and S4). To test whether EphA4 interacts *in vivo* with E-cadherin and ADAM10 at the IPC/OPC junction, we performed two types of *in situ* proximity ligation assays (Söderberg et al., 2006). In *EphA4*^*+/EGFP*^ animals, positive signals (detected as fluorescence red spots) revealed that EphA4^EGFP^ closely associated with E-cadherin and ADAM10, and that E-cadherin associated with ADAM10 at half-height of the PCs (Figure 5B). Of note, the loss of cytoplasmic domain in EphA4^EGFP^ did not abolish the interaction with ADAM10, which occurs constitutively through mutual extracellular domains (Janes et al., 2005). As expected, no positive signals were observed at apical extremities of the IPC/OPC junction, i.e., where PCs remain closely connected (Figure S5). Negative controls obtained by omitting one of the primary antibodies further confirmed that our proximity ligation assay signals were indicative of protein interactions (Figure 5C). In WT mice, positive signals revealed that EphA4 interacts with E-cadherin and ADAM10, and negative controls were obtained by pre-incubating the anti-EphA4 antibody with a blocking peptide (Figure S6). These results suggest that EphA4, E-cadherin, and ADAM10 form a complex that could be recognized and activated by ephrin-B2 across the PC junction to promote the cleavage of E-cadherin.

### ADAM10 Inhibition Prevents the Separation of PCs

**Figure 6:**
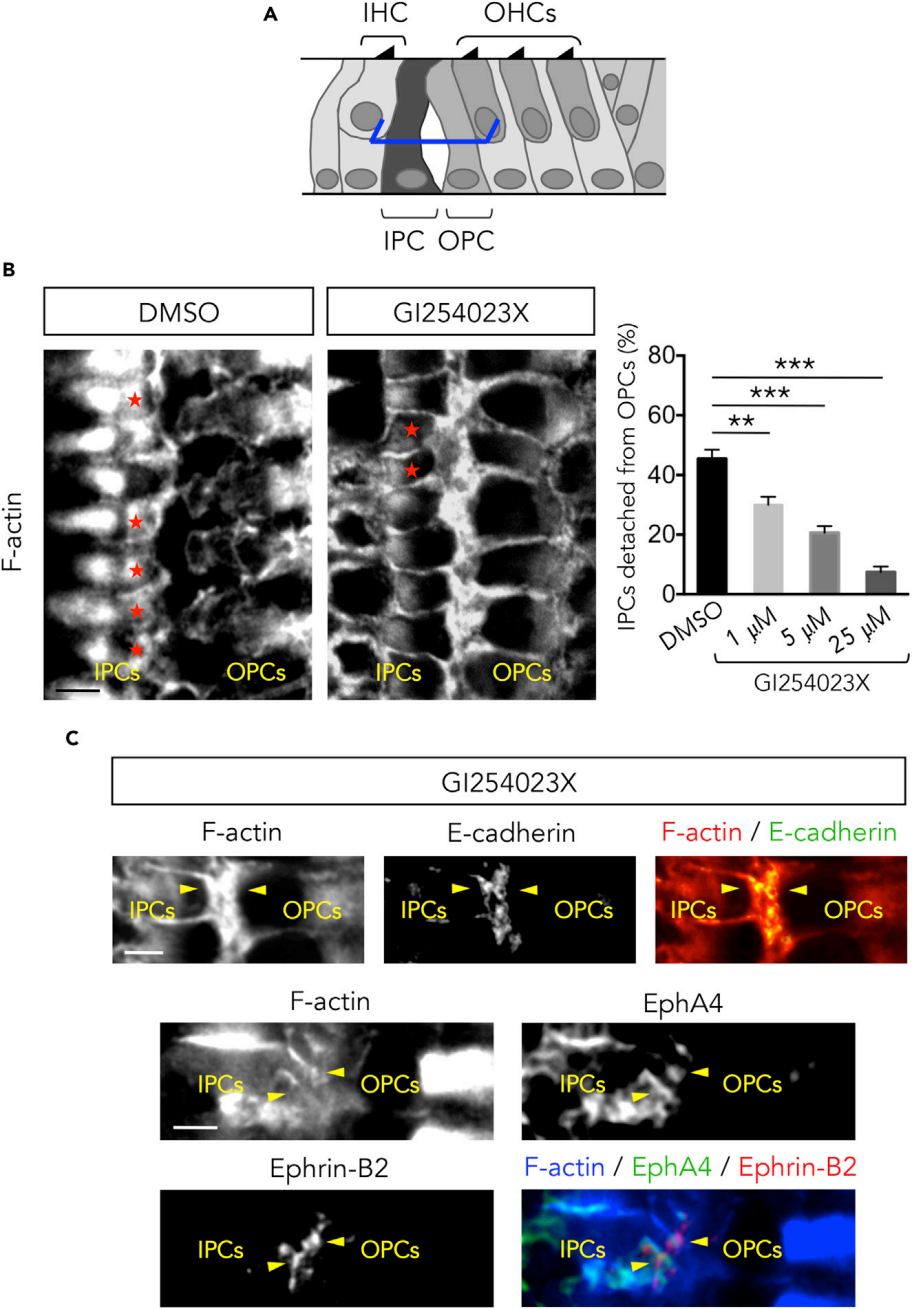
IPCs and OPCs Fail to Separate from Each Other in the Presence of ADAM10 Inhibitor (A) Schematic representation of a P8 organ of Corti showing the position at which level single confocal images were acquired (here at half-height of PCs, blue segments). (B) F-actin staining of organotypic culture treated with DMSO or with ADAM10 inhibitor (GI254023X). Organs of Corti from P2 mice were cultured for 6 days *in vitro*. Left panels: confocal images showing that, in control condition (DMSO), about half of the IPCs are separated from the OPCs (red asterisks). In the presence of GI254023X, few IPCs are fully detached from adjacent OPCs (red asterisks) and a large majority of PCs remain attached to each other. Right panel: the percentage of IPCs detached from OPCs is significantly decreased in the presence of GI254023X when compared with the control condition. This effect is concentration dependent. n = 300 IPCs from three independent experiments. (C) Upper panels: F-actin staining and E-cadherin immunolabeling showing that E-cadherin abnormally persists at the IPC/OPC junction (yellow arrowheads) in culture treated with GI254023X. Lower panels: F-actin staining and EphA4 and ephrin-B2 immunolabeling showing that the EphA4/ephrin-B2 complex persists at the IPC/OPC junction (yellow arrowheads) in culture treated with GI254023X. Data are presented as mean ± SEM. **p < 0.01, ***p < 0.001. Scale bars, 5 μm in (B) and (C). IHC, inner hair cell; IPC, inner pillar cell; OHC, outer hair cell; OPC, outer pillar cell.

To test whether ADAM10 cooperates with EphA4 to promote PC separation, we performed an organotypic *in vitro* assay aimed at blocking the endogenous activity of ADAM10 using GI254023X. We found that the percentage of IPCs entirely detached from the adjacent OPCs at half-height of the organ of Corti is significantly decreased in the presence of ADAM10 inhibitor (Figure 6B). In addition, we found that E-cadherin persists at the IPC/OPC junctions in treated organotypic culture (Figure 6C), suggesting that ADAM10 downregulates E-cadherin to promote PC detachment. Moreover, the EphA4/ephrin-B2 complex persists at the IPC/OPC junction in the presence of ADAM10 inhibitor, meaning that ADAM10 protease activity is also required for the cleavage of the Eph/ephrin complex and further cell-cell detachment, as previously described (Janes et al., 2005, Janes et al., 2009) (Figure 6D).

### Disruption of Adherens Junctions Rescues the EphA4 Loss of Function

**Figure 7:**
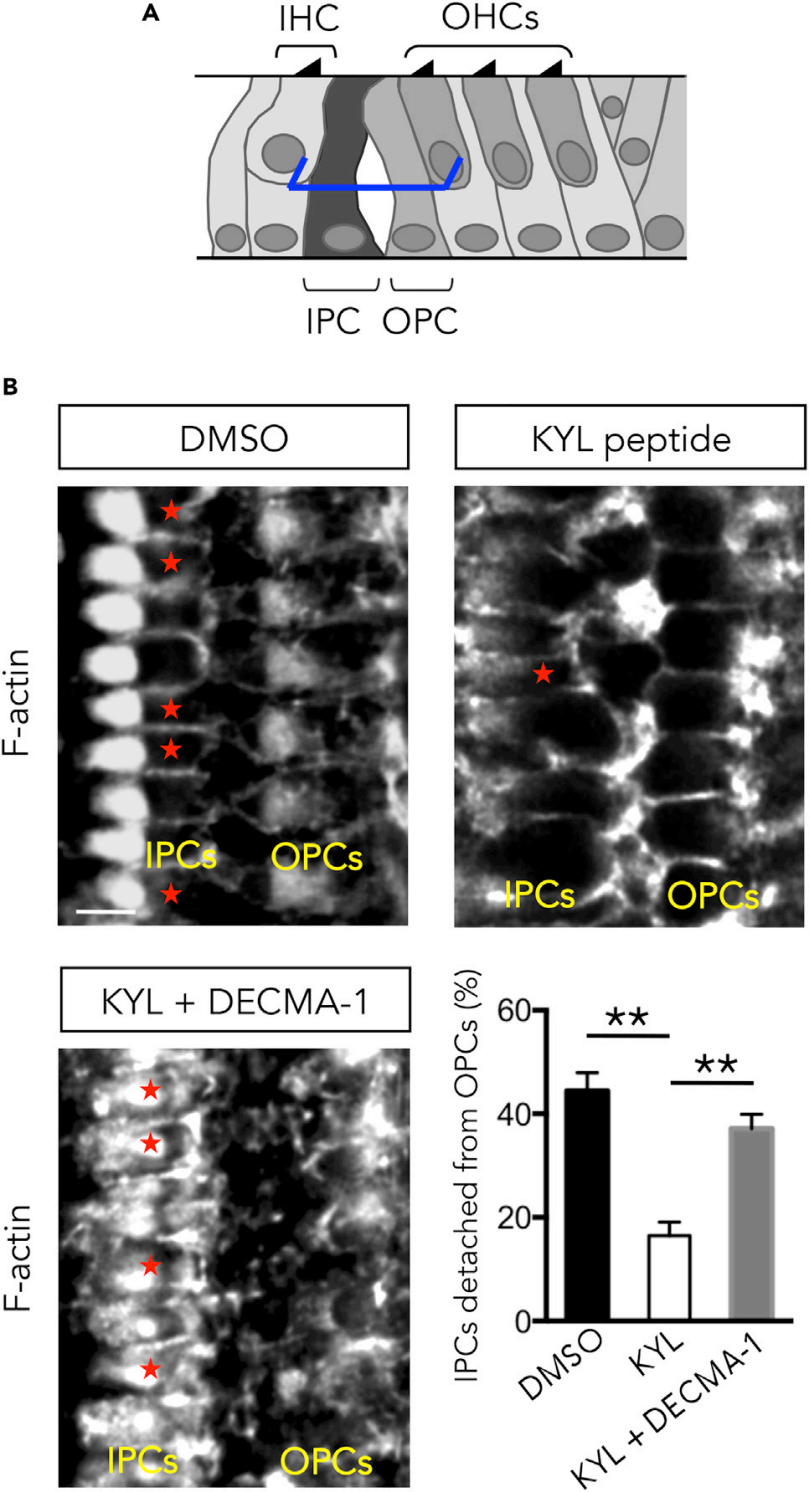
Disruption of Adherens Junctions Rescues the EphA4 Loss of Function (A) Schematic representation of a P8 organ of Corti showing the position at which level single confocal images were acquired (here at half-height of PCs, blue segments). (B) F-actin staining of organotypic culture treated with DMSO, with KYL peptide, or with KYL peptide and DECMA-1 mAb. Organs of Corti from P2 mice were cultured for 6 days *in vitro*. In control condition (DMSO), about half of the IPCs are separated from the OPCs (red asterisks). In the presence of KYL peptide, a few IPCs are fully separated from the adjacent OPCs (red asterisks) and a large majority of PCs remain attached to each other. The addition of DECMA-1 mAb to KYL peptide significantly rescues the percentage of IPCs detached from the OPCs. n = 300 IPCs from three independent experiments. Data are presented as mean ± SEM. **p < 0.01. Scale bar, 5 μm in (B). IHC, inner hair cell; IPC, inner pillar cell; OHC, outer hair cell; OPC, outer pillar cell.

Finally, we examined whether PC separation could be restored despite the loss of EphA4 activity. To this end, we performed a “two-step” organotypic *in vitro* assay. First, organs of Corti were pre-treated with KYL peptide to block the process of PC separation. Cultures were then incubated with an E-cadherin-neutralizing antibody, DECMA-1 mAb, aimed to promote the disruption of adherens junctions (Vestweber and Kemler, 1985). We found that E-cadherin downregulation efficiently rescues the EphA4 loss of function and restores the PC separation (Figure 7).

## Discussion

**Figure 8:**
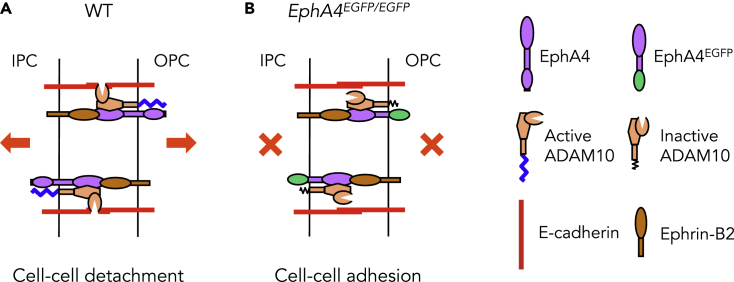
A Model for How Antiparallel EphA4 Forward Signaling Promotes the Disruption of Adherens Junctions (A) In WT mice, the *trans*-activation of EphA4 on both sides of the IPC/OPC junction results in a productive alignment with ADAM10 through cytoplasmic domains. This association mediates the correct orientation of the protease domain for cleavage of E-cadherin and further cell-cell detachment. (B) In mice in which the EphA4 cytoplasmic domain has been replaced by EGFP, ADAM10 cannot be *cis*-activated by EphA4 and correctly orientate its protease domain for cleavage of E-cadherin. As a consequence, IPCs and OPCs fail to separate from each other. IPC, inner pillar cell; OPC, outer pillar cell.

Here we show that two antiparallel EphA4 forward signals promote the destruction of E-cadherin-based adhesions between adjacent cochlear PCs. Our results support that EphA4, E-cadherin, and its sheddase ADAM10 form a complex that could be activated by ephrin-B2 across the PC junction to promote the cleavage of E-cadherin by ADAM10. Among EphA4-binding partners, ephrin-B2 and ephrin-B3 are especially involved in cell-cell separation (Rohani et al., 2014). As ephrin-B3 is apparently not expressed in cochlear PCs (Zhou et al., 2011), ephrin-B2 is the most likely partner of EphA4 to promote PC detachment. Previous data have shown that, upon binding, EphA3 clustering and auto-phosphorylation result in release of the intracellular tyrosine kinase domain away from the cell membrane into a conformation that facilitates the productive alignment with ADAM10 and the correct orientation of its protease domain (Janes et al., 2009). In agreement, our data suggest a model in which, upon binding in both directions across the PC junction, the *trans*-activation of EphA4 results in a productive association with ADAM10 through cytoplasmic domains. The *cis*-activation of ADAM10 by EphA4 leads to cleavage of E-cadherin and cell-cell detachment (Figure 8A). In contrast, in mice lacking the cytoplasmic domain of EphA4, ADAM10 cannot be *cis*-activated by EphA4 and fails to orientate its protease domain for the cleavage of E-cadherin. As a consequence, E-cadherin persists at the cell junction and the adjacent PCs fail to separate from each other (Figure 8B). Together, our results suggest a key role for antiparallel Eph receptor activation. Whereas a single Eph receptor forward signaling induces cell sorting through cleavage of E-cadherin (Solanas et al., 2011), two antiparallel Eph receptor forward signals promote cell-cell separation.

This mechanism of adherens junction downregulation by EphA4 might be extended to a broad range of physiological or pathological processes. In cancer with poor prognosis, EphA4 and E-cadherin appear to be regulated in opposite ways. On the one hand, the loss of E-cadherin expression is associated with tumor development, metastatic dissemination, and poor patient prognosis (Oka et al., 1993, Schipper et al., 1991, Umbas et al., 1994). On the other hand, EphA4 is overexpressed in a wide range of cancers (including glioblastoma, pancreatic, colorectal, gastric, prostate, and breast cancers) and is often associated with poor patient prognosis and shorter survival (Ashida et al., 2004, Brantley-Sieders et al., 2011, Fukai et al., 2008, Hachim et al., 2017, Iiizumi et al., 2006, Lin et al., 2017, Miyazaki et al., 2013, Oki et al., 2008, Oshima et al., 2008). Moreover, it has been shown that high EphA4 expression negatively correlates with metastasis-free survival (Brantley-Sieders et al., 2011, Lin et al., 2017, Miyazaki et al., 2013, Oshima et al., 2008). In this context, EphA4 promotes the motility and invasion of pancreatic cancer cells in part through the downregulation of E-cadherin (Liu et al., 2014). Loss of E-cadherin is a primary event in the initiation of epithelial-mesenchymal transition (EMT) (Onder et al., 2008, Thiery et al., 2009). Recent studies suggest that EMT particularly contributes to recurrent metastasis formation after chemotherapy (Fischer et al., 2015, Zheng et al., 2015). Interestingly, overexpression of EphA4 predicts a lesser degree of tumor regression after neoadjuvant chemoradiotherapy in rectal cancer (Lin et al., 2017). In addition, EphA4 signaling regulates the aggressive phenotype of irradiated colorectal cancer cells. Irradiation increases the activation of EphA4 in survivor colorectal cancer cells and promotes the internalization of an EphA4/E-cadherin complex, inducing cell-cell adhesion disruption (de Marcondes et al., 2016). Furthermore, EphA4 knockdown in the progeny of irradiated cells reduces the migratory and invasive potentials (de Marcondes et al., 2016). These findings have triggered numerous efforts that currently seek to develop pharmacological inhibitors of EphA4 for clinical use (Lamberto et al., 2012, Lamberto et al., 2014, Noberini et al., 2008, Schoonaert et al., 2017, Takano et al., 2015). At least as a regulator of adherens junctions, EphA4 now emerges as an attractive therapeutic target to prevent cancer cell dissemination and metastasis, including strategies aimed at overcoming chemo- and/or radioresistance.

### Limitations of the Study

Our results suggest that EphA4 forms a complex with E-cadherin and its sheddase ADAM10, which could be activated by ephrin-B2 across the PC junction to trigger the cleavage of E-cadherin. The first limitation concerns the real function of ephrin-B2 in this process, which was not addressed in this work. The second limitation concerns the presumed cleavage of E-cadherin by ADAM10, which was not directly proven in this study.

## Methods

All methods can be found in the accompanying Transparent Methods supplemental file.

## Supplemental Information

Document S1. Transparent Methods and Figures S1–S6

## References

[bib1] Ashida S., Nakagawa H., Katagiri T., Furihata M., Iiizumi M., Anazawa Y., Tsunoda T., Takata R., Kasahara K., Miki T. (2004). Molecular features of the transition from prostatic intraepithelial neoplasia (PIN) to prostate cancer: genome-wide gene-expression profiles of prostate cancers and PINs. Cancer Res..

[bib2] Bowden T.A., Aricescu A.R., Nettleship J.E., Siebold C., Rahman-Huq N., Owens R.J., Stuart D.I., Jones E.Y. (2009). Structural plasticity of eph receptor A4 facilitates cross-class ephrin signaling. Structure.

[bib3] Brantley-Sieders D.M., Jiang A., Sarma K., Badu-Nkansah A., Walter D.L., Shyr Y., Chen J. (2011). Eph/ephrin profiling in human breast cancer reveals significant associations between expression level and clinical outcome. PLoS One.

[bib4] Chen P., Segil N. (1999). p27(Kip1) links cell proliferation to morphogenesis in the developing organ of Corti. Development.

[bib5] Colvin J.S., Bohne B.A., Harding G.W., McEwen D.G., Ornitz D.M. (1996). Skeletal overgrowth and deafness in mice lacking fibroblast growth factor receptor 3. Nat. Genet..

[bib6] Defourny J., Mateo Sánchez S., Schoonaert L., Robberecht W., Davy A., Nguyen L., Malgrange B. (2015). Cochlear supporting cell transdifferentiation and integration into hair cell layers by inhibition of ephrin-B2 signalling. Nat. Commun..

[bib7] Defourny J., Poirrier A.L., Lallemend F., Mateo Sánchez S., Neef J., Vanderhaeghen P., Soriano E., Peuckert C., Kullander K., Fritzsch B. (2013). Ephrin-A5/EphA4 signalling controls specific afferent targeting to cochlear hair cells. Nat. Commun..

[bib8] de Marcondes P.G., Bastos L.G., de-Freitas-Junior J.C., Rocha M.R., Morgado-Diaz J.A. (2016). EphA4-mediated signaling regulates the aggressive phenotype of irradiation survivor colorectal cancer cells. Tumour Biol..

[bib9] Doetzlhofer A., Basch M.L., Ohyama T., Gessler M., Groves A.K., Segil N. (2009). Hey2 regulation by FGF provides a Notch-independent mechanism for maintaining pillar cell fate in the organ of Corti. Dev. Cell.

[bib10] Egea J., Nissen U.V., Dufour A., Sahin M., Greer P., Kullander K., Mrsic-Flogel T.D., Greenberg M.E., Kiehn O., Vanderhaeghen P. (2005). Regulation of EphA4 kinase activity is required for a subset of axon guidance decisions suggesting a key role for receptor clustering in Eph function. Neuron.

[bib11] Fischer K.R., Durrans A., Lee S., Sheng J., Li F., Wong S.T., Choi H., El Rayes T., Ryu S., Troeger J. (2015). Epithelial-to-mesenchymal transition is not required for lung metastasis but contributes to chemoresistance. Nature.

[bib12] Fukai J., Yokote H., Yamanaka R., Arao T., Nishio K., Itakura T. (2008). EphA4 promotes cell proliferation and migration through a novel EphA4-FGFR1 signaling pathway in the human glioma U251 cell line. Mol. Cancer Ther..

[bib13] Gale N.W., Holland S.J., Valenzuela D.M., Flenniken A., Pan L., Ryan T.E., Henkemeyer M., Strebhardt K., Hirai H., Wilkinson D.G. (1996). Eph receptors and ligands comprise two major specificity subclasses and are reciprocally compartmentalized during embryogenesis. Neuron.

[bib14] Grunwald I.C., Korte M., Adelmann G., Plueck A., Kullander K., Adams R.H., Frotscher M., Bonhoeffer T., Klein R. (2004). Hippocampal plasticity requires postsynaptic ephrinBs. Nat. Neurosci..

[bib15] Hachim I.Y., Villatoro M., Canaff L., Hachim M.Y., Boudreault J., Haiub H., Ali S., Lebrun J.J. (2017). Transforming growth factor-beta regulation of ephrin type-A receptor 4 signaling in breast cancer cellular migration. Sci. Rep..

[bib16] Iiizumi M., Hosokawa M., Takehara A., Chung S., Nakamura T., Katagiri T., Eguchi H., Ohigashi H., Ishikawa O., Nakamura Y. (2006). EphA4 receptor, overexpressed in pancreatic ductal adenocarcinoma, promotes cancer cell growth. Cancer Sci..

[bib17] Inoshita A., Iizuka T., Okamura H.O., Minekawa A., Kojima K., Furukawa M., Kusunoki T., Ikeda K. (2008). Postnatal development of the organ of Corti in dominant-negative Gjb2 transgenic mice. Neuroscience.

[bib18] Ito M., Spicer S.S., Schulte B.A. (1995). Cytological changes related to maturation of the organ of Corti and opening of Corti’s tunnel. Hear. Res..

[bib19] Jacques B.E., Montcouquiol M.E., Layman E.M., Lewandowski M., Kelley M.W. (2007). Fgf8 induces pillar cell fate and regulates cellular patterning in the mammalian cochlea. Development.

[bib20] Janes P.W., Saha N., Barton W.A., Kolev M.V., Wimmer-Kleikamp S.H., Nievergall E., Blobel C.P., Himanen J.P., Lackmann M., Nikolov D.B. (2005). Adam meets Eph: an ADAM substrate recognition module acts as a molecular switch for ephrin cleavage in trans. Cell.

[bib21] Janes P.W., Wimmer-Kleikamp S.H., Frangakis A.S., Treble K., Griesshaber B., Sabet O., Grabenbauer M., Ting A.Y., Saftig P., Bastiaens P.I. (2009). Cytoplasmic relaxation of active Eph controls ephrin shedding by ADAM10. PLoS Biol..

[bib22] Johnen N., Francart M.E., Thelen N., Cloes M., Thiry M. (2012). Evidence for a partial epithelial-mesenchymal transition in postnatal stages of rat auditory organ morphogenesis. Histochem. Cell Biol..

[bib23] Kania A., Klein R. (2016). Mechanisms of ephrin-Eph signalling in development, physiology and disease. Nat. Rev. Mol. Cell Biol..

[bib24] Karavitaki A.D., Mountain D.C. (2007). Evidence for outer hair cell driven oscillatory fluid flow in the tunnel of Corti. Biophys. J..

[bib25] Kullander K., Klein R. (2002). Mechanisms and functions of Eph and ephrin signalling. Nat. Rev. Mol. Cell Biol..

[bib26] Lamberto I., Lechtenberg B.C., Olson E.J., Mace P.D., Dawson P.E., Riedl S.J., Pasquale E.B. (2014). Development and structural analysis of a nanomolar cyclic peptide antagonist for the EphA4 receptor. ACS Chem. Biol..

[bib27] Lamberto I., Qin H., Noberini R., Premkumar L., Bourgin C., Riedl S.J., Song J., Pasquale E.B. (2012). Distinctive binding of three antagonistic peptides to the ephrin-binding pocket of the EphA4 receptor. Biochem. J..

[bib28] Lin C.Y., Lee Y.E., Tian Y.F., Sun D.P., Sheu M.J., Lin C.Y., Li C.F., Lee S.W., Lin L.C., Chang I.W. (2017). High expression of EphA4 predicted lesser degree of tumor regression after neoadjuvant chemoradiotherapy in rectal cancer. J. Cancer.

[bib29] Liu C., Huang H., Wang C., Kong Y., Zhang H. (2014). Involvement of ephrin receptor A4 in pancreatic cancer cell motility and invasion. Oncol. Lett..

[bib30] Maretzky T., Reiss K., Ludwig A., Buchholz J., Scholz F., Proksch E., de Strooper B., Hartmann D., Saftig P. (2005). ADAM10 mediates E-cadherin shedding and regulates epithelial cell-cell adhesion, migration, and beta-catenin translocation. Proc. Natl. Acad. Sci. U S A.

[bib31] Mellitzer G., Xu Q., Wilkinson D.G. (1999). Eph receptors and ephrins restrict cell intermingling and communication. Nature.

[bib32] Miyazaki K., Inokuchi M., Takagi Y., Kato K., Kojima K., Sugihara K. (2013). EphA4 is a prognostic factor in gastric cancer. BMC Clin. Pathol..

[bib33] Mueller K.L., Jacques B.E., Kelley M.W. (2002). Fibroblast growth factor signaling regulates pillar cell development in the organ of Corti. J. Neurosci..

[bib35] Noberini R., Koolpe M., Peddibhotla S., Dahl R., Su Y., Cosford N.D., Roth G.P., Pasquale E.B. (2008). Small molecules can selectively inhibit ephrin binding to the EphA4 and EphA2 receptors. J. Biol. Chem..

[bib36] Oka H., Shiozaki H., Kobayashi K., Inoue M., Tahara H., Kobayashi T., Takatsuka Y., Matsuyoshi N., Hirano S., Takeichi M. (1993). Expression of E-cadherin cell adhesion molecules in human breast cancer tissues and its relationship to metastasis. Cancer Res..

[bib37] Oki M., Yamamoto H., Taniguchi H., Adachi Y., Imai K., Shinomura Y. (2008). Overexpression of the receptor tyrosine kinase EphA4 in human gastric cancers. World J. Gastroenterol..

[bib38] Onder T.T., Gupta P.B., Mani S.A., Yang J., Lander E.S., Weinberg R.A. (2008). Loss of E-cadherin promotes metastasis via multiple downstream transcriptional pathways. Cancer Res..

[bib39] Oshima T., Akaike M., Yoshihara K., Shiozawa M., Yamamoto N., Sato T., Akihito N., Nagano Y., Fujii S., Kunisaki C. (2008). Overexpression of EphA4 gene and reduced expression of EphB2 gene correlates with liver metastasis in colorectal cancer. Int. J. Oncol..

[bib40] Peuckert C., Aresh B., Holenya P., Adams D., Sreedharan S., Porthin A., Andersson L., Pettersson H., Wölfl S., Klein R. (2016). Multimodal Eph/Ephrin signaling controls several phases of urogenital development. Kidney Int..

[bib41] Qin H., Noberini R., Huan X., Shi J., Pasquale E.B., Song J. (2010). Structural characterization of the EphA4-Ephrin-B2 complex reveals new features enabling Eph-ephrin binding promiscuity. J. Biol. Chem..

[bib42] Raphael Y., Altschuler R.A. (2003). Structure and innervation of the cochlea. Brain Res. Bull..

[bib43] Rohani N., Canty L., Luu O., Fagotto F., Winklbauer R. (2011). EphrinB/EphB signalling controls embryonic germ layer separation by contact-induced cell detachment. PLoS Biol..

[bib44] Rohani N., Parmeggiani A., Winklbauer R., Fagotto F. (2014). Variable combinations of specific ephrin ligand/Eph receptor pairs control embryonic tissue separation. PLoS Biol..

[bib45] Schipper J.H., Frixen U.H., Behrens J., Unger A., Jahnke K., Birchmeier W. (1991). E-cadherin expression in squamous cell carcinomas of head and neck: inverse correlation with tumor dedifferentiation and lymph node metastasis. Cancer Res..

[bib46] Schoonaert L., Rué L., Roucourt B., Timmers M., Little S., Chávez-Gutiérrez L., Dewilde M., Joyce P., Curnock A., Weber P. (2017). Identification and characterization of nanobodies targeting the EphA4 receptor. J. Biol. Chem..

[bib48] Söderberg O., Gullberg M., Jarvius M., Ridderstråle K., Leuchowius K.J., Jarvius J., Wester K., Hydbring P., Bahram F., Larsson L.G. (2006). Direct observation of individual endogenous protein complexes in situ by proximity ligation. Nat. Methods.

[bib49] Solanas G., Cortina C., Sevillano M., Batlle E. (2011). Cleavage of E-cadherin by ADAM10 mediates epithelial cell sorting downstream of EphB signalling. Nat. Cell Biol..

[bib50] Takano H., Nakamura T., Tsuchikawa T., Kushibiki T., Hontani K., Inoko K., Takahashi M., Sato S., Abe H., Takeuchi S. (2015). Inhibition of Eph receptor A4 by 2,5-dimethylpyrrolyl benzoic acid suppresses human pancreatic cancer growing orthotopically in nude mice. Oncotarget.

[bib51] Thiery J.P., Acloque H., Huang R.Y., Nieto M.A. (2009). Epithelial-mesenchymal transitions in development and disease. Cell.

[bib52] Umbas R., Isaacs W.B., Bringuier P.P., Schaafsma H.E., Karthaus H.F., Oosterhof G.O., Debruyne F.M., Schalken J.A. (1994). Decreased E-cadherin expression is associated with poor prognosis in patients with prostate cancer. Cancer Res..

[bib53] Vestweber D., Kemler R. (1985). Identification of a putative cell adhesion domain of uvomorulin. EMBO J..

[bib54] Whitlon D.S. (1993). E-cadherin in the mature and developing organ of Corti of the mouse. J. Neurocytol..

[bib55] Xu Q., Mellitzer G., Robinson V., Wilkinson D.G. (1999). In vivo cell sorting in complementary segmental domains mediated by Eph receptors and ephrins. Nature.

[bib56] Zheng X., Carstens J.L., Kim J., Scheible M., Kaye J., Sugimoto H., Wu C.C., LeBleu V.S., Kalluri R. (2015). Epithelial-to-mesenchymal transition is dispensable for metastasis but induces chemoresistance in pancreatic cancer. Nature.

[bib57] Zhou C.Q., Lee J., Henkemeyer M.J., Lee K.H. (2011). Disruption of ephrin B/Eph B interaction results in abnormal cochlear innervation patterns. Laryngoscope.

