# Supplementary material for: EphA4-ADAM10 Interplay Patterns the Cochlear Sensory Epithelium through Local Disruption of Adherens Junctions
Source: iScience. 2018 Dec 26;11:246–57. doi: 10.1016/j.isci.2018.12.017 (PMC6327856; doi:10.1016/j.isci.2018.12.017)
Supplement: Document S1. Transparent Methods and Figures S1–S6 [file mmc1.pdf]

ISCI, Volume *11*

Supplemental Information

EphA4-ADAM10 Interplay Patterns the Cochlear Sensory Epithelium through Local Disruption of Adherens Junctions

Jean Defourny, Christiane Peuckert, Klas Kullander, and Brigitte Malgrange

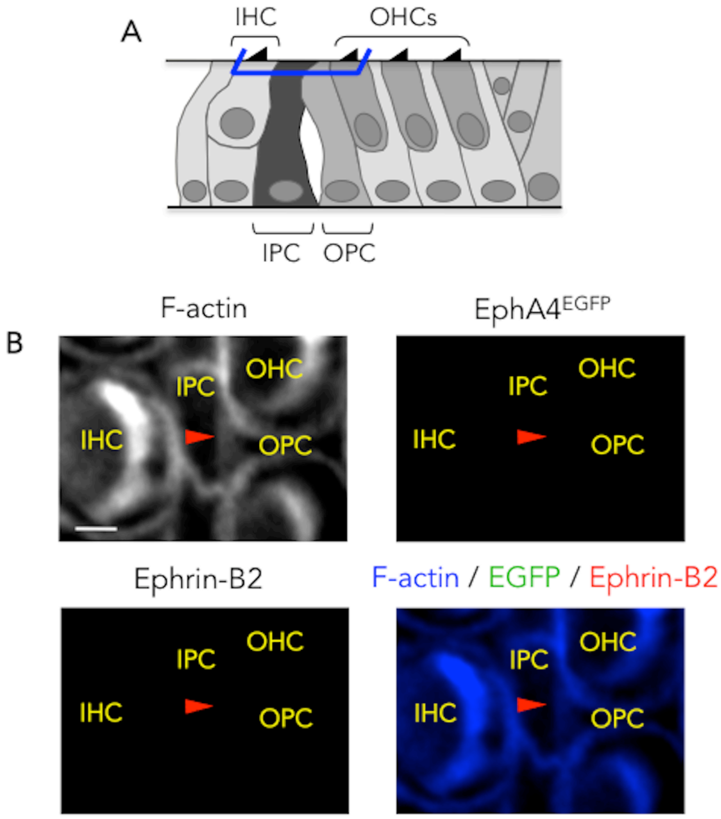

Figure S1. Neither EphA4 nor ephrin-B2 is expressed at the apical extremity of the IPC/OPC junction. Related to Figure 2. (**A**) Schematic representation of a P4 mouse organ of Corti showing the position at which level (here at the apical extremity of the PCs, blue segments) single confocal images were acquired. (**B**) F-actin staining and ephrin-B2 immunolabelling of a P4 *EphA4*$^{+/EGFP}$ whole-mount cochlea showing that neither EphA4^{EGFP} nor ephrin-B2 is present at the apical extremity of the IPC/OPC junction (red arrowhead). Scale bar represents 2 µm. IHC = inner hair cell, IPC = inner pillar cell, OHC = outer hair cell, OPC = outer pillar cell.

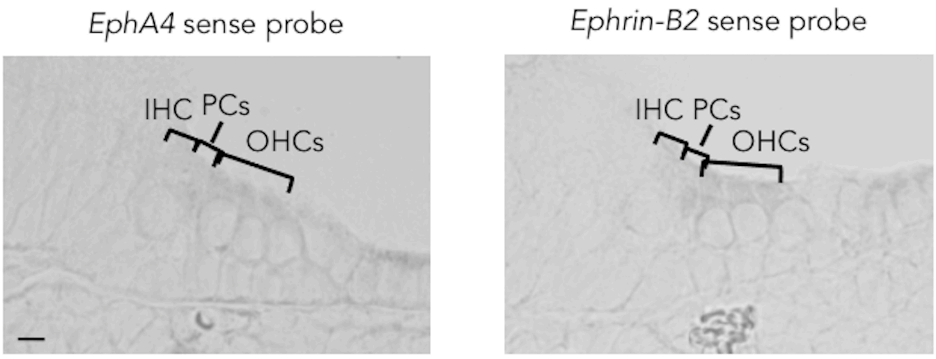

Figure S2. In situ hybridization negative controls. Related to Figure 2. ISH negative controls were obtained using *EphA4* and *ephrin-B2* sense probes on transversal sections of P4 organ of Corti. Scale bar represents 5 µm. IHC = inner hair cell, OHCs = outer hair cells, PCs = pillar cells.

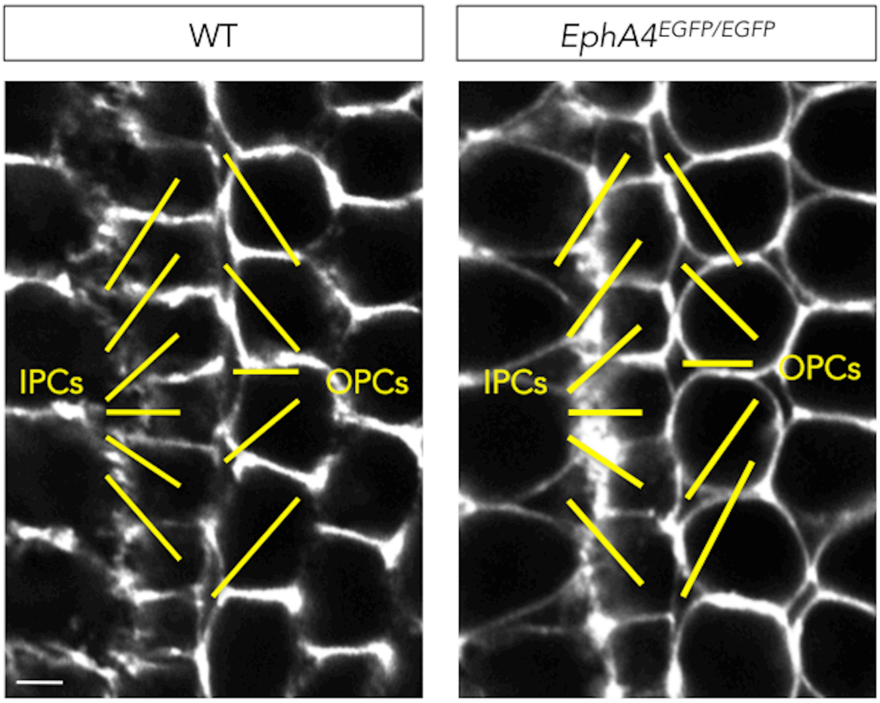

Figure S3. ***EphA4*$^{EGFP/EGFP}$ PCs do not display any defects in early postnatal stages. Related to Figure 3.** F-actin staining of P2 whole-mount WT and *EphA4*$^{EGFP/EGFP}$ cochleae showing that *EphA4*$^{EGFP/EGFP}$ PCs do not display any defects as compared to WT PCs. Scale bar represents 5 µm. IPCs = inner pillar cells, OPCs = outer pillar cells.

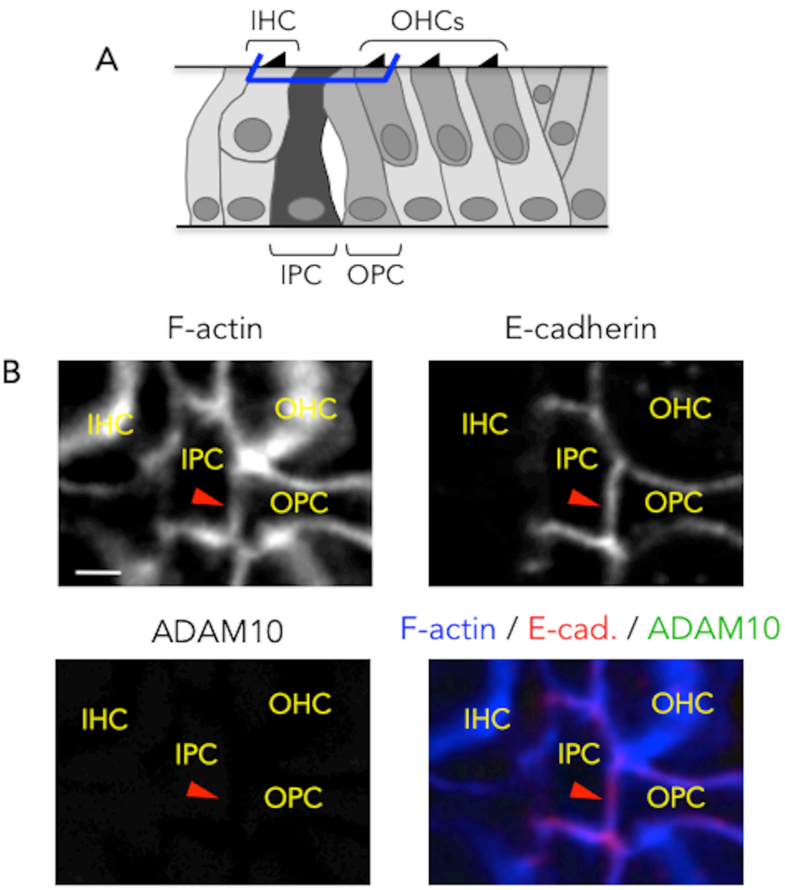

Figure S4. E-cadherin but not ADAM10 is present at the apical extremity of the IPC/OPC junction. Related to Figure 4. (**A**) Schematic representation of a P6 mouse organ of Corti showing the position at which level (here at the apical extremity of the PCs, blue segments) single confocal images were acquired. (**B**) F-actin staining, E-cadherin and ADAM10 immunolabelling of a P6 WT whole-mount cochlea showing that E-cadherin but not ADAM10 is present at the apical extremity of the IPC/OPC junction (red arrowhead). Scale bar represents 2 µm. IHC = inner hair cell, IPC = inner pillar cell, OHC = outer hair cell, OPC = outer pillar cell.

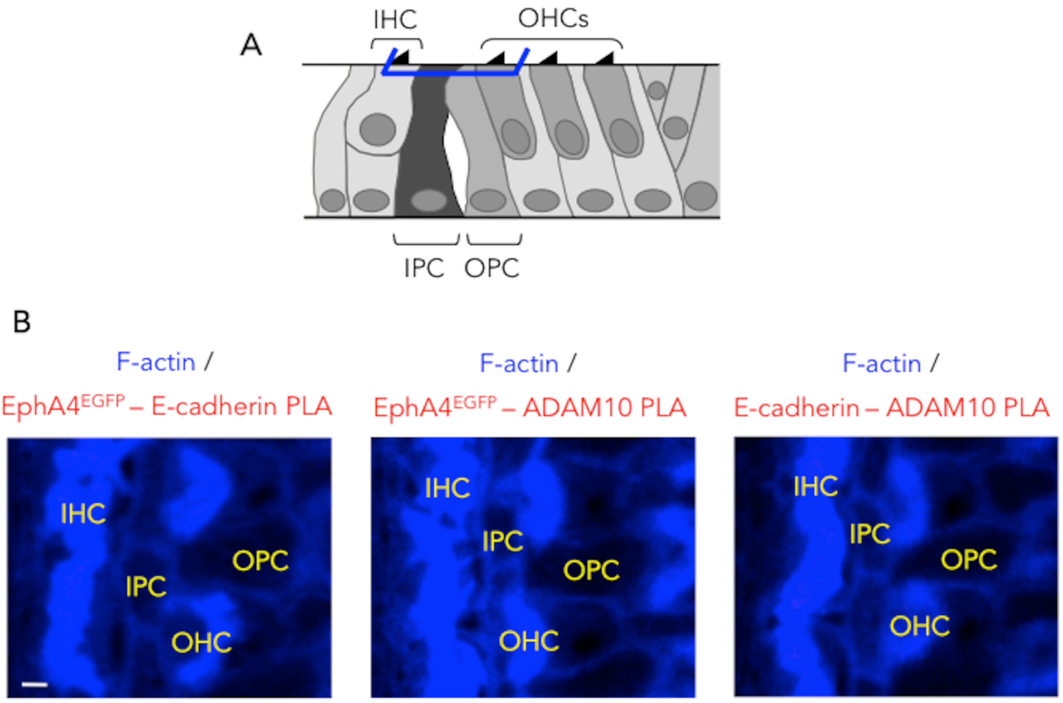

Figure S5. No EphA4^{EGFP} - E-cadherin, EphA4^{EGFP} - ADAM10 or ADAM10 - E-cadherin PLA signals are observed at the apical extremity of the PC junction. Related to Figure 5. (**A**) Schematic representation of a P6 mouse organ of Corti showing the position at which level (here at the apical extremity of the PCs, blue segments) single confocal images were acquired. (**B**) *In situ* proximity ligation assay showing that EphA4^{EGFP} does not interact with E-cadherin or ADAM10, and E-cadherin does not interact with ADAM10 at the apical extremity of the IPC/OPC junction. Scale bar represents 2 μm. IHC = inner hair cell, IPC = inner pillar cell, OHC = outer hair cell, OPC = outer pillar cell, PLA = proximity ligation assay.

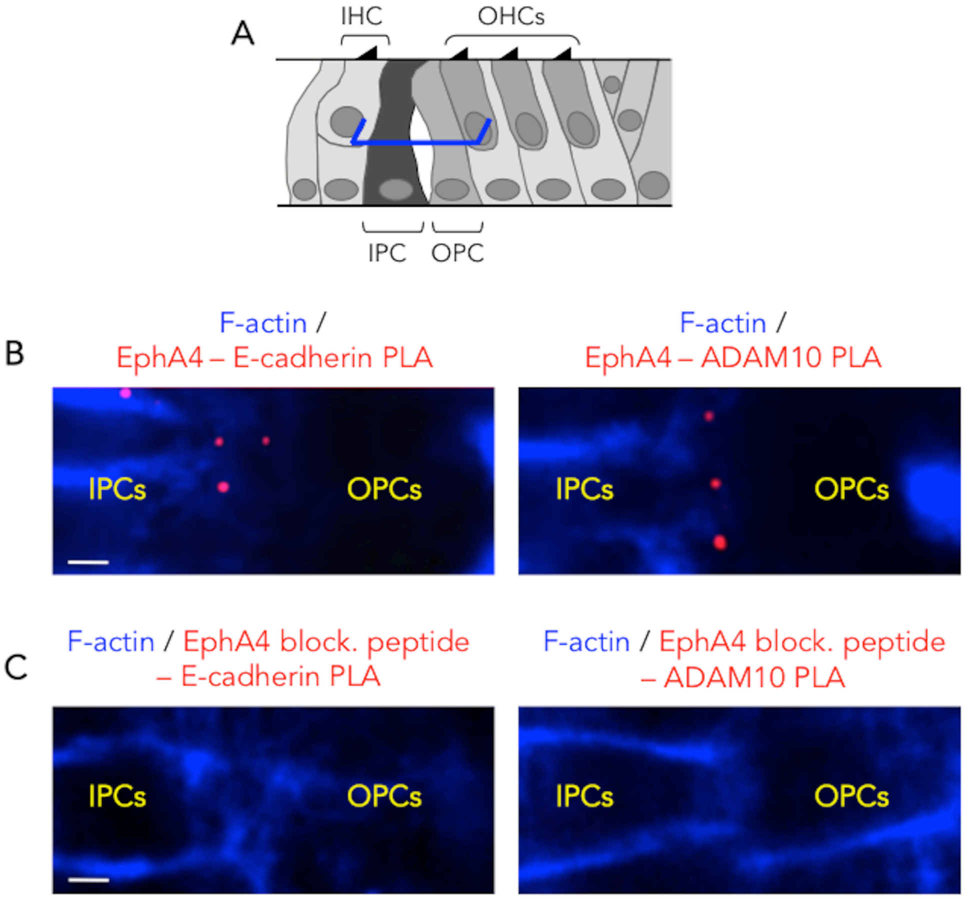

Figure S6. EphA4 interacts with E-cadherin and ADAM10 at the IPC/OPC junction in WT mice. Related to Figures 4 and 5. (**A**) Schematic representation of a P6 mouse organ of Corti showing the position at which level single confocal images were acquired (here at half-height of PCs, blue segments). (**B**) *In situ* proximity ligation assay showing that EphA4 interacts with E-cadherin and ADAM10. (**C**) Proximity ligation assay negative controls were obtained by pre-incubating the anti-EphA4 antibody with a blocking peptide. Scale bars represent 2 µm. IPCs = inner pillar cells, OPCs = outer pillar cells, PLA = proximity ligation assay.

Transparent Methods

Animals

WT, *EphA4*$^{+/EGFP}$ and *EphA4*$^{EGFP/EGFP}$ littermates were obtained from heterozygous crosses and genotype was determined by PCR as previously described (Grunwald et al., 2004). Mice were group-housed in the animal facility of the University of Liège under standard conditions with food and water *ad libitum* and were maintained on a 12-hour light/dark cycle. All animals were taken care of in accordance with the declaration of Helsinki and following the guidelines of the Belgian ministry of agriculture in agreement with EC laboratory animal care and use regulation (2010/63/UE, 22 September 2010).

Tissue processing and immunolabelling

Postnatal cochleae were fixed for 24 hours in 4% PFA at 4° C. Samples were then washed and decalcified for 2 or 3 days (0.1 M EDTA in PBS). Permeabilization and blockage of unspecific binding sites were performed by 30 minutes of incubation at room temperature (RT) in blocking solution (0.25% gelatin and 0.3% Triton X-100 in PBS). Whole-mount cochleae were incubated overnight at 4° C with primary antibodies directed against GFP (rat monoclonal IgG2a, 1:250, Gentaur Genprice, London, UK), EphA4 (mouse monoclonal IgG2b; 1:50; Santa Cruz Biotechnology, Dallas, TX, USA, AB_10843811), ephrin-B2 (goat polyclonal IgG, 1:250, R&D Systems, Minneapolis, MN, USA, AB_2095679; rabbit monoclonal IgG, 1:250, Abcam, Cambridge, UK), E-cadherin (rabbit monoclonal IgG, 1:250, Abcam, Cambridge, UK, AB_562059; mouse monoclonal IgG1, 1:100, Abnova, Taipei City, Taiwan, AB_1671631) and ADAM10 (rabbit polyclonal IgG, 1:100, Abcam, Cambridge, UK, AB_302747). After washings in PBS, tissues were incubated for 1h at RT in blocking solution containing Cy5-conjugated goat anti-rabbit or anti-goat IgGs secondary antibodies (Jackson Immunoresearch Laboratories, Suffolk, UK) and tetramethyl rhodamine isothiocyanate (TRITC)-phalloidin (1:1000, Sigma Aldrich, St Louis, MO, USA) as F-actin marker. Finally, tissues were washed in PBS, mounted and coverslipped using VectaShield Hard Set mounting medium containing DAPI (Vector Laboratories, Burlingame, CA, USA). Slides were stored in the dark at 4°C.

Confocal microscopy, image analysis and quantification

Fluorescence pictures were acquired using the Olympus Fluoview FV1000 confocal system equipped with the Olympus IX81 inverted microscope (Olympus Europa GmbH, Germany).

Single confocal plane were acquired at half-height and at apical extremity of the cochlear PCs. To reconstruct the whole organ of Corti in orthogonal projection, fields were acquired using Z-scan with a step of 0.5µm between each confocal plane. For comparison between genotypes and culture conditions, all preparations were analyzed at the same time, using the same acquisition parameters.

On whole-mount two-week-old cochleae (P14), we considered IPCs as fully detached from OPCs when no F-actin-positive material was visible between each other. Corresponding regions were quantified at apical, middle and basal turns of cochleae from WT and *EphA4*$^{EGFP/EGFP}$ mice. The same criteria were used to compare organotypic culture conditions. Values were summed and expressed as a percentage of IPCs that are completely detached from OPCs. Data were statistically analysed using Student's t-test, or using one-way ANOVA followed by Dunnett's post-test. *P* values less than 0.05 were considered significant (**p<0.01; ***p<0.001).

In situ *proximity ligation assay*

In order to characterize endogenous protein interactions, we used the Duolink *in situ* proximity ligation assay reagent (Olink Biosciences, Uppsala, Sweden). Whole-mount cochleae were treated and handled as for immunolabelling (see above). Three combinations of primary antibodies were used for incubation of *EphA4*$^{+/EGFP}$ tissues overnight at 4°C: anti-GFP and anti-E-cadherin (rabbit antibody), anti-GFP and anti-ADAM10, anti-E-cadherin (mouse antibody) and anti-ADAM10. For the assays using rat anti-GFP, whole-mount cochleae were incubated for 1h at room temperature with mouse anti-rat antibody. Negative controls were obtained by omitting one of the two primary antibodies. Two combinations of primary antibodies were used for incubation of WT tissues: anti-EphA4 and anti-E-cadherin (rabbit antibody), anti-EphA4 and anti-ADAM10. Negative controls were obtained by pre-incubating the anti-EphA4 antibody (1:50) with a blocking peptide (1:5). Oligo-labelled anti-mouse plus and anti-rabbit minus probes were then used as recommended by the manufacturer. Cochleae were labelled using FluoProbes 647H – Phalloidin (1:100, Cheshire Sciences, Chester, UK) then mounted using VectaShield Hard Set mounting medium. Proximity ligation assay images (fluorescent spots) combined to F-actin staining were acquired as for immunolabelling above.

In vitro *organotypic assay*

Organs of Corti were isolated from P2 mice and cultured for 6 days onto Millicell Culture Insert (Millipore) as previously described (Defourny et al., 2013). Organotypic cultures were incubated for 24 h with dimethyl sulfoxide (DMSO, vehicle) or ADAM10 inhibitor GI254023X (1, 5 and 25 μM ; Sigma-Aldrich). GI254023X is a potent and selective ADAM10 metalloproteinase inhibitor with 100-fold selectivity for the α-secretase ADAM10 over ADAM17 (TACE). Otherwise, organotypic cultures were pre-incubated for 6 h with KYL peptide (50 μM ; Tocris Bioscience, Bristol, UK) and incubated for another 12 h with DECMA-1 mAb (rat monoclonal IgG1 ; 20 μg/mL ; Abcam ; AB_298118).

RNA in situ *hybridization*

In situ hybridization was performed using digoxigenin-labelled *EphA4* and *ephrin-B2* riboprobes (Nieto et al., 1992 ; Smith et al., 1997) as previously described (Defourny et al., 2013). Sections were air-dried, washed in PBS and post-fixed in paraformaldehyde. After treatment with 100 mM triethanolamine, pH 8, acetylated (by adding dropwise acetic anhydride 0.25%) while rocking 15 min at room temperature (RT), slides were washed three times in PBS-0.1% Tween-20 and pre-hybridized in pre-warmed hybridization cocktail 50% formamide (Amresco) at least 60 min at 70 °C. Afterwards, sections were hybridized with 800 ng ml^{-1} RNA probes overnight at 70 °C. Sections were then washed twice in pre-warmed washing buffer (50% formamide, 2 x sodium salt citrate, 0.1% Tween-20) for 60 min at 70 °C. Slides were pre-incubated for 1 h in blocking solution (Tris-Saline buffer (100 mM Tris pH 7.5; 150 mM NaCl) containing 10% Normal Goat Serum (Dako)) at RT and subsequently incubated overnight at 4 °C with anti-digoxigenin antibody coupled to alkaline phosphatase (1:2,000, Roche Applied Science) diluted in blocking solution. After three washes with Tris-Saline buffer, sections were finally overlaid with 200 ml filtered NBT/BCIP (Nitro Blue Tetrazolium/5-Bromo-4-Chloro-3-Indolyl Phosphate) - 0.1% Tween-20 solution (Sigma-Aldrich) between coverslips in dark and at RT until the signal appears. Slides were then washed, post-fixed and mounted in aquamount medium (BDH laboratories).

Supplemental References

Nieto, M.A., Gilardi-Hebenstreit, P., Charnay, P., and Wilkinson, D.G. (1992). A receptor protein tyrosine kinase implicated in the segmental patterning of the hindbrain and mesoderm. Development *116*, 1137-1150.

Smith, A., Robinson V., Patel, K., and Wilkinson D. G. (1997). The EphA4 and EphB1 receptor tyrosine kinases and ephrin-B2 ligand regulate targeted migration of branchial neural crest cells. Curr. Biol. *7*, 561-570.
